# P-624. Serotyping of Streptococcus pneumoniae and Clinical Outcomes Among Hospitalized Patients in Louisville, Kentucky

**DOI:** 10.1093/ofid/ofaf695.837

**Published:** 2026-01-11

**Authors:** Forest W Arnold, Leslie A Parrish, Subathra Marimuthu, Vidyulata Salunkhe, Daniya Sheikh, Nataly Pazmino, Jafir Wakeel, Girish Madishetty, Hamza Mehmood, Spozhmai Hewadmai, Biplab Adhikari, Hassan Cheema, Hussnain Cheema, Imad Majeed, Hamza Asif, Steven Gootee, T’shura Ali

**Affiliations:** University of Louisville School of Medicine, Louisville, KY; University of Louisville, Division of Infectious Diseases, Louisville, Kentucky; School of Medicine, University of Louisville, Louisville, Kentucky; University of Louisville School of Medicine, Louisville, KY; University of Louisville School of Medicine, Louisville, KY; University of Louisville, Division of Infectious Diseases, Louisville, Kentucky; University of Louisville, Louisville, Kentucky; University of Louisville, Division of Infectious Diseases, Louisville, Kentucky; University of Louisville, Division of Infectious Diseases, Louisville, Kentucky; University of Louisville, Division of Infectious Diseases, Louisville, Kentucky; University of Louisville, Division of Infectious Diseases, Louisville, Kentucky; University of Louisville, Division of Infectious Diseases, Louisville, Kentucky; University of Louisville, Division of Infectious Diseases, Louisville, Kentucky; University of Louisville, Division of Infectious Diseases, Louisville, Kentucky; University of Louisville Hospital, Louisville, KY; University of Louisville School of Medicine, Louisville, KY; University of Louisville School of Medicine, Louisville, KY

## Abstract

**Background:**

*Streptococcus pneumoniae* serotypes 3, 6A, 6B, 9N and 19F often cause severe disease. The pneumococcal conjugate vaccines PCV-20 and PCV-21 cover 10 shared serotypes, along with 10 and 11 additional unique serotypes, respectively. The association between serotype coverage and severity of disease remains poorly understood.

Serotype distribution of Streptococcus pneumoniae Among Hospitalized Patients

**Figure:**
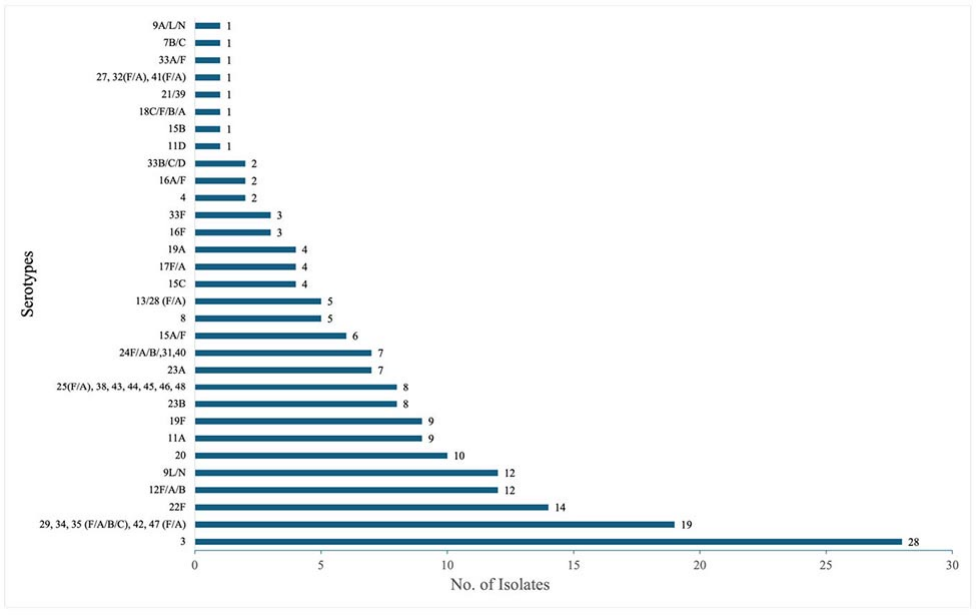

**Figure:**
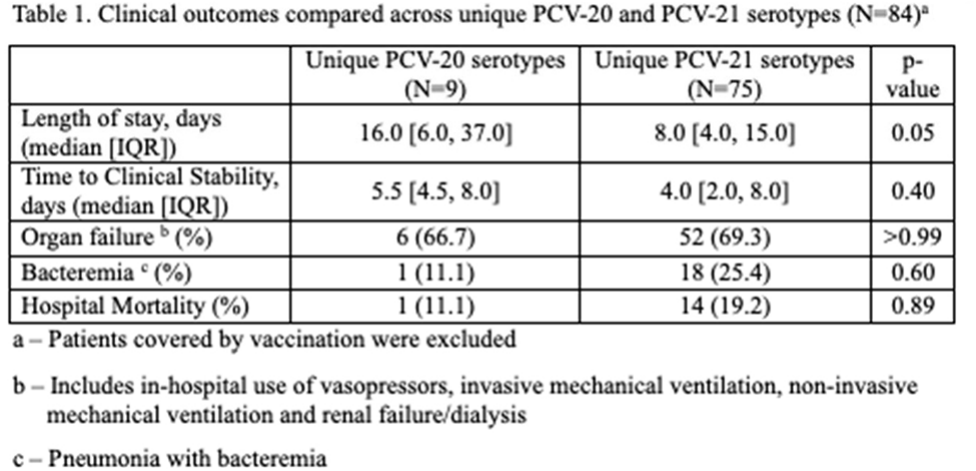

**Methods:**

This was a secondary analysis of patients with *S. pneumoniae* pneumonia. Serotype was determined by Quellung reaction and PCR. Demographics were collected, including vaccination status, as well as time to clinical stability, length of stay, bacteremia (invasive disease), organ failure and mortality. Patients were grouped by serotype and categorized based on PCV-20 or PCV-21 vaccine serotype. Clinical outcomes were compared between each group. For the PCV-20 serotype category, clinical outcomes were also compared by vaccination status.

**Results:**

A total of 191 patients were included: 89 (47%) had a serotype covered by the PCV-20 vaccine and 151 (70%) had a serotype covered by the PCV-21 vaccine. Of the 89 patients with PCV-20 serotypes, 13 (15%) were unique to the PCV-20 vaccine, and of the 151 patients with PCV-21 serotypes, 83 (55%) were unique to the PCV-21 vaccine. No significant differences in clinical outcomes were observed between unique serotype categories; however, PCV-20 serotypes tended to result in longer time to stability (5.5 vs. 4 days) and hospital stay (16 vs. 8 days), while PCV-21 serotypes showed a trend toward higher rates of bacteremia (25% vs. 11%), organ failure (69% vs. 67%), and mortality (19% vs. 11%). Among those with any serotype covered by the PCV-20 vaccine, clinical outcomes did not differ significantly by vaccination status, though unvaccinated individuals showed non-significant trends toward worse outcomes—longer time to stability (5 vs. 4 days), length of stay (8 vs. 5 days), and higher mortality (13% vs. 11%).

**Conclusion:**

More patients had a serotype covered by a unique serotype of PCV-21 vaccine than PCV-20 vaccine, but clinical severity was comparable. Among the PCV-20 vaccine group, vaccination status did not result in significant improvement in outcomes. However, observed trends suggest potential variations in disease severity that warrant further investigation.

**Disclosures:**

Forest W. Arnold, DO, MSc, Gilead Sciences: Grant/Research Support Leslie A. Parrish, Ph.D., Gilead Sciences: Grant/Research Support Subathra Marimuthu, MS, Gilead Sciences: Grant/Research Support Vidyulata Salunkhe, MBBS, MPH, Gilead Sciences: Grant/Research Support Daniya Sheikh, MD, Gilead Sciences: Grant/Research Support Nataly Pazmino, MD, Gilead Sciences: Grant/Research Support Jafir Wakeel, MBBS, Gilead Sciences: Grant/Research Support Girish Madishetty, MD, Gilead Sciences: Grant/Research Support Hamza Mehmood, MD, Gilead Sciences: Grant/Research Support Spozhmai Hewadmai, MD, Gilead Sciences: Grant/Research Support Biplab Adhikari, MD, Gilead Sciences: Grant/Research Support Hassan Cheema, MD, Gilead Sciences: Grant/Research Support Hussnain Cheema, MD, Gilead Sciences: Grant/Research Support Imad Majeed, MD, Gilead Sciences: Grant/Research Support Steven Gootee, MHI, Gilead Sciences: Grant/Research Support T'shura Ali, PhD, MPH, Gilead Sciences: Grant/Research Support

